# Tumor-Associated Macrophages and Collagen Remodeling in Mammary Carcinomas: A Comparative Analysis in Dogs and Humans

**DOI:** 10.3390/ijms26146928

**Published:** 2025-07-18

**Authors:** Ana Paula Vargas Garcia, Marisa Salvi, Luana Aparecida Reis, Bárbara Regina Melo Ribeiro, Cristiana Buzelin Nunes, Ana Maria de Paula, Geovanni Dantas Cassali

**Affiliations:** 1 Department of General Pathology, Institute of Biological Sciences, Federal University of Minas Gerais, Av. Pres. Antônio Carlos, 6627, Pampulha, Belo Horizonte 31270-901, MG, Brazil; apvg@ufmg.br (A.P.V.G.); salvi@ufmg.br (M.S.); 2 Department of Physics, Institute of Exact Sciences, Federal University of Minas Gerais, Av. Pres. Antônio Carlos, 6627, Pampulha, Belo Horizonte 31270-901, MG, Brazil; luana.apreis@gmail.com (L.A.R.); barbara2rmelo@gmail.com (B.R.M.R.); ana@fisica.ufmg.br (A.M.d.P.); 3 Department of Anatomic Pathology, Faculty of Medicine, Federal University of Minas Gerais, Av. Prof. Alfredo Balena, 190, Santa Efigênia, Belo Horizonte 30130-100, MG, Brazil; cristianabnunes@gmail.com

**Keywords:** collagen fibers, tumor-associated macrophages, breast cancer, tumor microenvironment, comparative oncology

## Abstract

The tumor microenvironment (TME) plays a central role in cancer progression, with tumor-associated macrophages (TAMs) and extracellular matrix (ECM) components such as collagen being key modulators of invasiveness and immune regulation. Although macrophage infiltration and ECM remodeling are well-documented individually, their coordinated contribution to mammary carcinoma aggressiveness remains underexplored, particularly in comparative oncology models. This study analyzed 117 mammary carcinoma samples—59 from dogs and 58 from women—using immunohistochemistry, immunofluorescence, and second-harmonic-generation (SHG) microscopy. We quantified TAM density and phenotype (CD206, iNOS, and S100A8/A9), assessed collagen fiber organization, and examined correlations with clinical–pathological variables and overall survival. Increased TAM infiltration was associated with a higher histological grade, aggressive molecular subtypes, enhanced cell proliferation, and shortened survival in dogs. High TAM density also correlated with decreased collagen fiber length and increased alignment, suggesting active immune–matrix remodeling in aggressive tumors. Macrophage phenotyping revealed heterogeneous populations, with CD206^+^ cells predominating in high-grade tumors, while S100A8/A9^+^/iNOS^+^ phenotypes were enriched in less aggressive subtypes. The findings were consistent across species, reinforcing the relevance of canine models. Our results identify macrophage–collagen interactions as critical determinants of tumor aggressiveness in mammary carcinomas. This study bridges comparative oncology and translational research by proposing immune–ECM signatures as potential prognostic biomarkers and therapeutic targets. These insights contribute to the advancement of molecular oncology in Brazil by supporting innovative strategies that integrate immune modulation and matrix-targeted interventions in breast cancer.

## 1. Introduction

Breast cancer remains one of the most prevalent malignancies and a leading cause of cancer-related deaths among women worldwide. In 2020 alone, over 2.3 million new cases were diagnosed, and more than 685,000 women died from the disease globally, underscoring the urgent need for improved prognostic tools and treatment strategies [1]. Despite the advances in early detection, molecular profiling, and systemic therapies, tumor recurrence, metastatic dissemination, and therapeutic resistance continue to pose major clinical challenges [2,3,4,5]. These limitations have driven increasing interest in the tumor microenvironment (TME) as a key modulator of cancer behavior and as a potential therapeutic target.

The TME is a complex and dynamic ecosystem composed not only of malignant epithelial cells but also of stromal fibroblasts, endothelial cells, immune infiltrates, and extracellular matrix (ECM) components [5,6,7,8,9]. Among the ECM constituents, collagen fibers are particularly abundant and strongly influence tissue architecture, stiffness, and cellular behavior [2,4,10,11]. Traditionally regarded as passive structural scaffolds, collagen fibers are now recognized as active participants in tumor progression, capable of regulating cell adhesion, migration, and mechanotransduction [12,13,14,15]. Alterations in collagen fiber alignment, density, and cross-linking are associated with increased tumor invasiveness, immune evasion, and worse prognoses in several malignancies, including breast cancer [15,16,17,18,19,20,21,22,23,24,25,26,27,28,29,30,31,32,33,34,35,36,37,38,39,40].

Importantly, the ECM serves not only as a physical barrier or conduit but as a dynamic signaling platform. The biophysical properties of collagen networks—such as fiber tension, topography, and porosity—have been implicated in the regulation of gene expression and activation of mechanosensitive pathways in both tumor and immune cells [41,42,43,44,45]. These properties can modulate integrin clustering, focal adhesion turnover, and YAP/TAZ signaling, influencing cellular proliferation and differentiation.

In parallel, the immune compartment of the TME—particularly tumor-associated macrophages (TAMs)—has emerged as a critical determinant of tumor fate [46,47,48]. These highly plastic myeloid cells can adopt pro-tumoral (M2-like) or anti-tumoral (M1-like) phenotypes depending on the prevailing microenvironmental cues [42,49,50]. M2-polarized TAMs secrete immunosuppressive cytokines, promote angiogenesis, facilitate ECM remodeling, and support tumor cell invasion [48,51,52]. Their infiltration has been strongly correlated with adverse outcomes, metastatic relapse, and resistance to conventional therapies [52,53].

Recent studies have revealed that TAMs and collagen fibers engage in bidirectional crosstalk that actively shapes the TME. Collagen organization can influence macrophage recruitment and polarization [54,55], while TAMs remodel the ECM via the secretion of matrix metalloproteinases (MMPs), lysyl oxidase (LOX), and cathepsins [56,57]. This dynamic interaction contributes to the formation of permissive niches for tumor expansion and immune escape. However, despite its growing relevance, this interplay remains underexplored, particularly in comparative models that capture the heterogeneity of human disease.

Comparative oncology has emerged as a promising strategy to bridge preclinical and clinical research by leveraging spontaneous tumors in companion animals. Canine mammary tumors (CMTs) represent an especially relevant model due to their high incidence, histopathological diversity, and clinical progression patterns that mirror those of human breast cancer [58,59,60] Dogs share environmental exposures, hormonal cycles, and immune responses with humans, and their tumors arise naturally in immunocompetent hosts, offering advantages over genetically engineered murine models.

In this context, the present study aimed to evaluate the relationship between macrophage infiltration and collagen fiber architecture in mammary carcinomas of dogs and women. By combining histopathological assessment, immunohistochemistry, multiphoton imaging, and survival analysis, we sought to (1) identify correlations between TAM density and tumor aggressiveness; (2) characterize macrophage phenotypes in situ; and (3) determine the prognostic relevance of TAM–ECM interactions in both species. Given the histological and molecular similarities between canine and human breast cancer, this study also provides translational insights into potential immunomodulatory and ECM-targeted therapeutic strategies.

## 2. Results

### 2.1. Characterization of the Analyzed Samples

The clinicopathological characteristics of the human and canine mammary carcinoma cohorts are summarized in Table 1, including the histological subtypes, patient age, TNM staging, histological grades, molecular subtypes, and survival data. This stratification allows for cross-species comparison and supports the analysis of tumor-associated macrophage (TAM) infiltration patterns across clinically relevant subgroups.

### 2.2. Histopathological Profiles of the Mammary Carcinomas Evaluated

The histological subtypes evaluated in this study encompassed a spectrum of mammary carcinomas with distinct morphological and biological profiles. In human samples, tubular carcinoma (TC) and invasive carcinoma grade I (ICgI) were representative of well-differentiated low-grade neoplasms. TC, in particular, is characterized by uniform small ducts lined by a single layer of epithelial cells with minimal nuclear atypia and rare mitotic figures, often associated with excellent prognosis. ICgI presents slightly more architectural complexity but still maintains glandular organization and a low proliferative index [61].

Conversely, invasive carcinoma grade III (ICgIII) and invasive micropapillary carcinoma (IMC) represent aggressive phenotypes. ICgIII exhibits high mitotic activity, marked nuclear pleomorphism, loss of tubule formation, and often necrosis, aligning with rapid clinical progression. IMC, although rare, is distinguished by small nests of tumor cells surrounded by clear stromal spaces mimicking lymphatic channels, facilitating early metastasis, particularly via lymphovascular invasion [61,62,63].

A similar morphological spectrum was observed in canine tumors [64]. Carcinoma in mixed tumors (CMT), one of the most common subtypes in dogs, showed abundant stromal and myoepithelial components with intermingled epithelial elements, generally linked to better prognosis [24,64,65,66]. Carcinosarcomas (CSs) and carcinomas with solid arrangement (CSAs), on the other hand, exhibited densely cellular sheets of pleomorphic epithelial cells, high mitotic indices, and frequent stromal invasion [64,66,67,68,69]. IMCs in dogs mirrored their human counterparts in both morphology and clinical aggressiveness, reinforcing their translational relevance.

This cross-species histopathological alignment provided a robust foundation to investigate the infiltration and role of tumor-associated macrophages (TAMs) within the tumor microenvironment (TME). Stratifying tumors into prognostic subgroups based on morphological criteria enabled meaningful correlation with macrophage density and ECM organization, and supported the hypothesis of TAM–ECM interaction as a common modulator of tumor behavior.

**Table 1:** Clinical characteristics of human and canine mammary carcinoma cohorts.

Species	Histological Type	n	Age (years)	Staging					Grade			Subtype				Survival ^a^ (mo/days)
				**I**	**II**	**III**	**IV**	**V**	**I**	**II**	**III**	**HR+/Ki67 < 20%**	**HR+/Ki67 > 20%**	**HER2+**	**HR-/HER2-**	
Human	tubular carcinoma (TC)	4	71.3	2	2	0	0	0	4	0	0	4	0	0	0	69.0
Human	invasive carcinoma grade I (ICgI)	16	68.4	5	7	0	3	1	16	0	0	13	3	0	0	76.3
Human	invasive carcinoma grade III (ICgIII)	21	59.6	5	2	2	10	2	0	0	21	2	12	4	3	62.1
Human	invasive micropapillary carcinoma (IMC)	17	52.3	4	3	1	9	0	3	8	2	3	8	6	0	52.3
Canine	carcinoma in mixed tumors (CMT)	14	11.0	7	3	4	0	0	11	2	1	13	1	0	0	1116.2
Canine	carcinosarcoma (CS)	6	8.5	0	0	4	0	2	0	0	6	2	4	0	0	321.8
Canine	invasive micropapillary carcinoma (IMC)	16	10.8	0	1	2	10	3	2	9	5	1	7	3	5	293.9
Canine	carcinoma with solid arrangement (CSA)	23	11.8	5	4	4	9	1	9	10	4	3	17	2	1	279.5

Comparative clinical and histopathological features of mammary carcinoma in human and canine subjects. The table summarizes the distribution of histological types, age, tumor stage (TNM), histological grade, molecular subtypes, and survival outcomes. The parallel structure enables cross-species analysis and supports the use of canine mammary carcinoma as a translational model for aggressive human breast cancer subtypes. Survival is shown in months (humans) and days (dogs). ^a^ Survival is shown in months (mo) for human subjects and days for canine subjects.

### 2.3. Increased TAM Infiltration Is Associated with Tumors of Poor Prognosis

**Figure 1:**
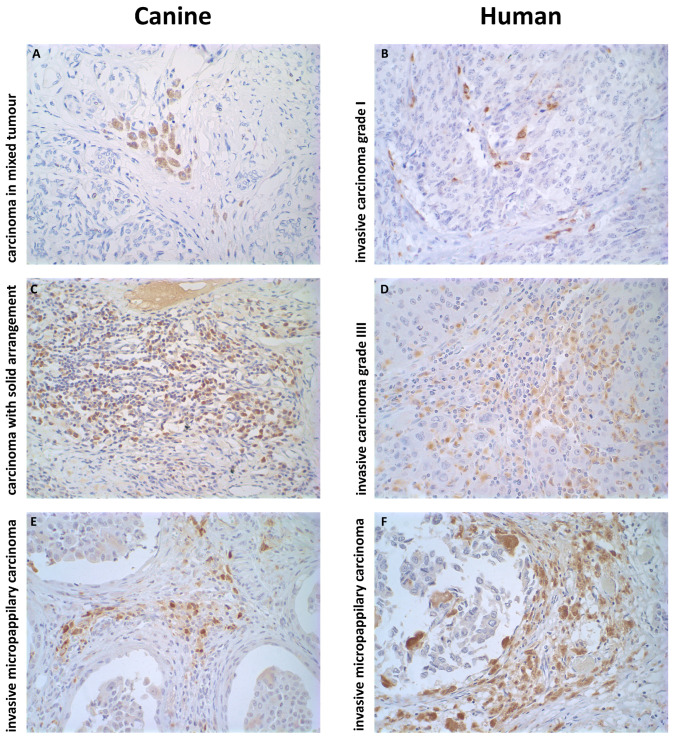
Immunohistochemical staining of CD68^+^ tumor-associated macrophages (TAMs) in representative canine and human mammary carcinomas. Panels illustrate macrophage distribution and density in (**A**) canine carcinoma in mixed tumors (CMT), (**B**) human invasive carcinoma grade I (ICgI), (**C**) canine solid carcinoma (SC), (**D**) human invasive carcinoma grade III (ICgIII), (**E**) canine invasive micropapillary carcinoma (IMC), and (**F**) human IMC. Tumors were selected based on histomorphological similarity and prognostic equivalence across species. CMT and ICgI represent well-differentiated low-grade subtypes, while SC and ICgIII are poorly differentiated high-grade carcinomas. IMC was included due to its aggressive behavior and shared architectural features in both species. A progressive increase in TAM infiltration is observed in more aggressive subtypes, supporting their association with tumor progression. Images acquired using 40× objective (scale bar = 100 μm) (**A**–**F**).

To investigate the spatial distribution and prognostic implications of tumor-associated macrophages (TAMs), we analyzed a spectrum of mammary carcinoma subtypes with varying degrees of malignancy in both canine and human specimens. Figure 1 presents representative immunohistochemical images of CD68^+^ TAM infiltration across well-differentiated and poorly differentiated tumor types.

In the canine cohort, carcinoma in mixed tumors (CMT) was selected as a prototype of a low-grade well-differentiated tumor with relatively indolent behavior and organized glandular architecture. Its human counterpart was invasive carcinoma grade I (ICgI), which also exhibits low proliferative activity and favorable prognosis. In contrast, solid carcinoma (SC) and carcinoma with solid arrangement (CSA) in dogs were aligned with grade III invasive carcinoma (ICgIII) in humans, reflecting high-grade poorly differentiated tumors characterized by cellular atypia, disorganized architecture, and frequent mitotic figures. Invasive micropapillary carcinoma (IMC), known for its aggressive clinical course, high rates of lymphovascular invasion, and tendency for early metastasis, was included in both species due to its distinctive histology and translational relevance.

These cross-species pairings were supported by previous studies comparing mammary tumor grading systems and their prognostic associations across dogs and humans [59,60,70]. While species-specific differences in nomenclature persist, these alignments provide a coherent framework for comparative oncology and enable meaningful investigation into immune–stromal interactions.

**Figure 2:**
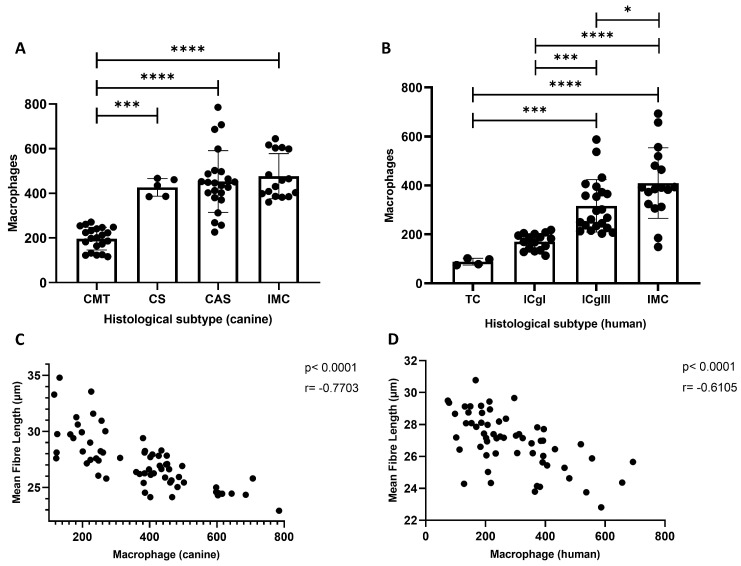
Quantification of tumor-associated macrophage (TAM) infiltration across different histological subtypes of canine (**A**) and human (**B**) mammary carcinomas. Boxplots represent the median and interquartile range (IQR) of macrophage counts per tumor. Statistically significant differences between subtypes are indicated. Panels (**C**,**D**) show the negative correlation between TAM density and collagen fiber length in canine (**C**) and human (**D**) samples, supporting a link between macrophage infiltration and extracellular matrix remodeling in aggressive tumors. * *p* < 0.05; *** *p* < 0.001; **** *p* < 0.0001).

Figure 1 illustrates clear differences in TAM distribution across tumor subtypes. In low-grade CMT and ICgI (panels A and B), CD68^+^ cells are sparse and perivascular, while, in high-grade SC, CSA, ICgIII, and IMC (panels C–F), TAMs are more abundant, frequently infiltrating tumor nests and the surrounding stromal compartments. These visual observations were quantitatively validated using image analysis across the entire cohort, as shown in Figure 2A,B.

Quantitative comparisons revealed that aggressive tumor subtypes consistently exhibited higher TAM infiltration. In the canine group, TAM density was significantly increased in CSA, SC, and IMC compared to CMT (p<0.001). Likewise, in the human cohort, ICgIII and IMC showed elevated macrophage counts relative to ICgI and tubular carcinoma (TC) (p<0.001). Statistical comparisons were conducted using the Kruskal–Wallis test followed by Dunn’s post hoc test.

Notably, TAM infiltration was not uniformly distributed even within high-grade tumors. We observed substantial intra-group heterogeneity in TAM density within ICgIII and IMC cases, suggesting the existence of immune microenvironmental heterogeneity within histologically similar lesions. This may reflect distinct immunoediting trajectories or tumor-specific recruitment of macrophages [51,52]. The high density of TAMs in aggressive subtypes is consistent with their known role in promoting tumor progression via the secretion of matrix metalloproteinases (MMPs), angiogenic factors (e.g., VEGF), and immunosuppressive cytokines [47,49,57]. This heterogeneity may have prognostic and therapeutic relevance, particularly in the context of emerging macrophage-targeted therapies that depend on the immune composition of individual tumors.

The findings further support the concept that TAMs are not merely passive bystanders but active participants in shaping the tumor microenvironment, particularly in poorly differentiated and invasive tumors. These results also reinforce the notion that histological grade alone may underestimate the biological complexity of tumor immune landscapes.

Altogether, this analysis reveals a consistent relationship between increased TAM infiltration and tumor aggressiveness in both canine and human mammary carcinomas. These observations underscore the value of macrophage quantification as a potential prognostic tool and provide a biologically plausible rationale for therapeutic targeting of TAMs in aggressive breast cancer subtypes.

### 2.4. Carcinomas with Shorter Collagen Fibers Have the Highest TAM Infiltration

Figure 2C,D show strong negative correlations between TAM infiltration and collagen fiber length in both canine (r = −0.7703, p<0.0001) and human (r = −0.6105, p<0.0001) mammary carcinomas. These findings suggest that higher macrophage densities are consistently associated with disorganized extracellular matrices characterized by shorter collagen fibers.

This relationship is biologically plausible as TAMs have been shown to promote matrix remodeling through the secretion of enzymes such as matrix metalloproteinase-9 (MMP-9) and lysyl oxidase (LOX), both of which contribute to collagen degradation, cross-linking, and fiber shortening [57,71] Conversely, changes in the physical properties of the ECM—including increased stiffness and altered fiber orientation—can activate integrin-dependent signaling pathways that attract and polarize macrophages toward an M2-like tumor-promoting phenotype [42,45,72]. Thus, the correlation observed here may reflect a bidirectional feedback loop between innate immune activation and ECM remodeling.

Furthermore, shorter collagen fibers have previously been associated with enhanced tumor invasiveness, increased interstitial fluid pressure, and reduced T cell infiltration [14,17]. In this context, the presence of abundant TAMs within a matrix-rich but disorganized stroma may serve to further exclude adaptive immune effectors, promoting an immunosuppressive niche that facilitates tumor progression.

Taken together, these results reinforce the role of ECM architecture as both a regulator and readout of tumor immunobiology. The consistent correlation across species supports the translational relevance of canine models for studying TAM–ECM interactions and suggests that fiber length may represent a morphometric biomarker of microenvironmental reprogramming in breast cancer.

### 2.5. More Aggressive Carcinomas Exhibit Higher TAM Infiltration

**Figure 3:**
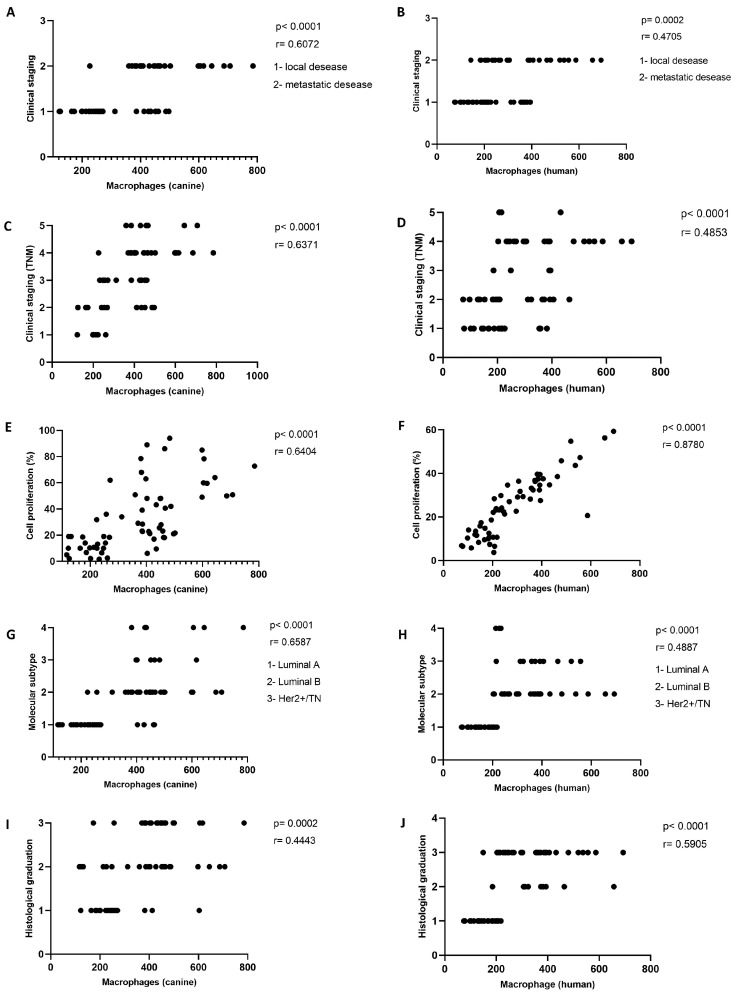
Spearman’s correlation analyses between tumor-associated macrophage (TAM) infiltration and clinicopathological parameters in canine (**A**,**C**,**E**,**G**,**I**) and human (**B**,**D**,**F**,**H**,**J**) mammary carcinomas. Significant positive correlations were observed between TAM density and clinical stage (**A**–**D**), cell proliferation index (**E**,**F**), molecular subtype classification (**G**,**H**), and histological grade (**I**,**J**), indicating that increased TAM infiltration is associated with more aggressive tumor features across both species.

To investigate the impact of tumor-associated macrophage (TAM) infiltration on the biological behavior of mammary carcinomas, we analyzed its relationship with key clinicopathological features in both canine and human specimens. Specifically, we assessed correlations between TAM density and clinical stage, histological grade, proliferative activity, and molecular subtype, as illustrated in Figure 3. Immunohistochemical analysis of CD68^+^ macrophages was performed on 58 human and 59 canine tumor samples.

Figure 3 demonstrates that TAM infiltration positively correlates with indicators of tumor aggressiveness across both species. Higher macrophage density was consistently associated with advanced clinical stage (Figure 3A,B), increased proliferation index (Figure 3E,F), more aggressive molecular subtypes (Figure 3G,H), and higher histological grade (Figure 3I,J). These findings are consistent with previous studies in human oncology that have identified TAMs as key components of an immunosuppressive microenvironment, particularly in high-grade and triple-negative breast cancers [46,48,53].

In the canine cohort, high TAM infiltration was observed in tumors classified as grade II or III, as well as those presenting regional lymph node involvement or distant metastases. Similar patterns were evident in the human cases, with macrophage accumulation particularly prominent in grade III carcinomas and HER2-overexpressing or triple-negative subtypes. These subtypes are known for their poor prognosis and resistance to conventional hormone therapies, and are frequently associated with elevated expression of angiogenic and ECM-remodeling factors [51,52].

The proliferation index, evaluated via Ki-67 immunostaining, also showed a significant positive correlation with TAM density. This relationship reinforces the hypothesis that TAMs support tumor growth by promoting mitogenic signaling and enabling immune evasion [42,49]. Furthermore, macrophage infiltration in both species correlated with molecular subtypes linked to more aggressive clinical behavior. While canine mammary tumors do not always conform to the molecular subclassification used in human breast cancer, cross-species parallels—such as ER-/PR- tumors and HER2 overexpression—provide valuable translational insights [70,73].

It is worth noting that, although HER2 status is a robust prognostic and therapeutic marker in human oncology, its significance in canine mammary carcinoma remains uncertain. Divergent results in the literature may reflect methodological differences, interspecies molecular variations, or tumor heterogeneity [74]. Nevertheless, our findings suggest that macrophage recruitment occurs preferentially in biologically aggressive tumors regardless of species-specific molecular classification nuances.

Overall, the data support the notion that TAM density could serve as a surrogate marker of tumor aggressiveness in both veterinary and human oncology. Moreover, given the increasingly recognized role of TAMs in driving immunosuppression, angiogenesis, and extracellular matrix remodeling, their quantification could inform both prognostic stratification and the development of macrophage-targeted therapies. Importantly, the consistent trends observed across species lend further weight to the relevance of comparative oncology as a platform for biomarker discovery and therapeutic innovation.

### 2.6. TAM Infiltration Stratifies Survival in Canine and Human Mammary Carcinomas

To assess the prognostic significance of tumor-associated macrophage (TAM) infiltration, we performed Kaplan–Meier survival analyses in both canine and human mammary carcinoma cohorts. In the canine group, we used the median TAM count (400 cells) as an unbiased cut-off, independent of histological subtype, clinical stage, histological grade, proliferative index, or molecular profile. The tumors were stratified into low (<400 macrophages) and high (≥400 macrophages) infiltration groups. Survival status was coded as 0 for animals alive at the end of follow-up and 1 for those who died due to mammary carcinoma.

**Figure 4:**
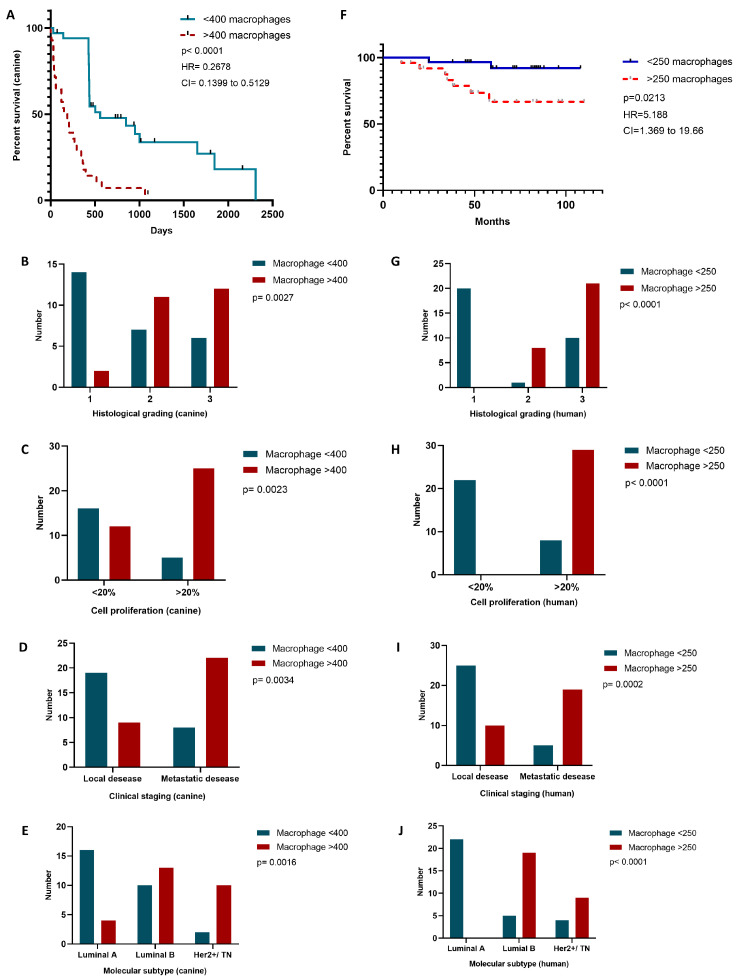
Tumor-associated macrophage (TAM) burden predicts survival and correlates with aggressive clinicopathological features in both canine and human mammary carcinomas. (**A**) Kaplan–Meier survival curve for dogs stratified by TAM infiltration (cut-off: 400 cells). High infiltration (≥400 macrophages, dashed red line) is associated with significantly shorter median survival (168 days) compared to low infiltration (<400 macrophages, solid blue line; 558 days). (**B**–**E**) Clinical and pathological features stratified by TAM burden in the canine cohort: tumor grade (**B**), proliferative index (**C**), clinical stage (**D**), and molecular subtype (**E**). High TAM infiltration is significantly associated with grade III tumors, Ki-67 > 20%, advanced clinical stage, and non-luminal subtypes. (**F**) Kaplan–Meier survival curve for humans stratified by TAM infiltration (cut-off: 250 cells). High infiltration (≥250 macrophages, dashed red line) correlates with reduced overall survival. (**G**–**J**) Clinicopathological associations in the human cohort: tumor grade (**G**), proliferative index (**H**), clinical stage (**I**), and molecular subtype (**J**). Similar to dogs, high TAM burden is significantly associated with more aggressive disease features. *p*-values were calculated using log-rank tests for survival analyses and chi-squared tests for categorical variables.

The canine Kaplan–Meier curve (Figure 4A) revealed a pronounced difference in survival outcomes between the two groups. Those dogs with high TAM infiltration (dashed red line) exhibited a significantly shorter median survival of 168 days compared to 558 days in the low-infiltration group (solid blue line). The difference was statistically significant (log-rank test, p<0.0001), with a hazard ratio (HR) of 3.734 and a 95% confidence interval (CI) of 1.950–7.150, indicating that macrophage burden is a strong predictor of clinical outcome in this model.

In the human cohort, a similar analysis was conducted using a TAM count cut-off of 250 cells, determined based on the distribution of macrophage infiltration across the cases. The patients were stratified into low (<250 macrophages) and high (≥250 macrophages) infiltration groups. Despite a limited number of events and substantial censoring, the survival analysis (Figure 4F) demonstrated a significant difference between the groups. High TAM infiltration was associated with reduced overall survival (log-rank test, p=0.0213), with an HR of 5.188 (95% CI: 1.369–19.66), suggesting that macrophage density bears prognostic relevance in human breast cancer.

Together, these findings support the role of TAM burden as a cross-species prognostic marker, reinforcing the translational potential of the canine model in evaluating tumor microenvironmental determinants of clinical outcome.

Importantly, these findings suggest that TAM infiltration reflects more than just tumor histopathology or clinical stage; it likely captures underlying immunological and stromal processes that actively drive disease progression. TAMs are known to promote tumor aggressiveness through diverse mechanisms, including the induction of angiogenesis, suppression of anti-tumor immune responses, and remodeling of the extracellular matrix [48,51,57]. Together, these processes shape a microenvironment permissive to tumor growth, invasion, and metastasis.

Our data demonstrate that high TAM infiltration is significantly associated with reduced overall survival in both canine and human mammary carcinomas (Figure 4A,F). These findings extend the prior clinical observations in human breast cancer—particularly in the triple-negative and HER2-positive subtypes [52,53]—by validating macrophage density as an independent prognostic factor. The parallel survival patterns observed across species reinforce the translational relevance of the canine model for investigating immune–stromal interactions in aggressive breast cancer subtypes.

To further explore the clinical and pathological associations of TAM burden, we stratified both cohorts by histological grade, proliferative index, clinical stage, and molecular subtype (Figure 4B–E for canine, and Figure 4G–J for human samples).

In the canine cohort, high TAM infiltration (>400 macrophages) was significantly associated with aggressive pathological features. Dogs with high macrophage counts were more likely to have poorly differentiated tumors (grade III, p=0.0027; Figure 4B), high cellular proliferation indices (>20%, p=0.0023; Figure 4C), advanced clinical staging with metastatic disease (p=0.0034; Figure 4D), and non-luminal molecular subtypes (Luminal B or HER2+/TN, p=0.0016; Figure 4E).

A similar pattern was observed in the human cohort. Those patients with high TAM infiltration (>250 macrophages) exhibited a higher frequency of grade III tumors (p<0.0001; Figure 4G), increased proliferative activity (p<0.0001; Figure 4H), metastatic disease at diagnosis (p=0.0002; Figure 4I), and were predominantly classified as Luminal B or HER2+/TN subtypes (p<0.0001; Figure 4J).

These results reinforce the association between elevated TAM burden and aggressive clinicopathological features in both species, further supporting its role as a marker of poor prognosis in mammary carcinomas.

### 2.7. Intratumoral TAMs Express M1 and M2 Markers Concurrently

After confirming that higher macrophage infiltration was associated with increased tumor aggressiveness and poorer prognosis in both humans and dogs—and significantly reduced survival in the canine cohort—we sought to characterize the phenotypic polarization of intratumoral TAMs. While the classical M1/M2 paradigm posits a dichotomy between pro-inflammatory, tumor-suppressive (M1) and anti-inflammatory, tumor-promoting (M2) macrophage states, increasing evidence suggests that TAMs in vivo may exhibit hybrid or intermediate phenotypes depending on microenvironmental cues [42,50,75].

**Figure 5:**
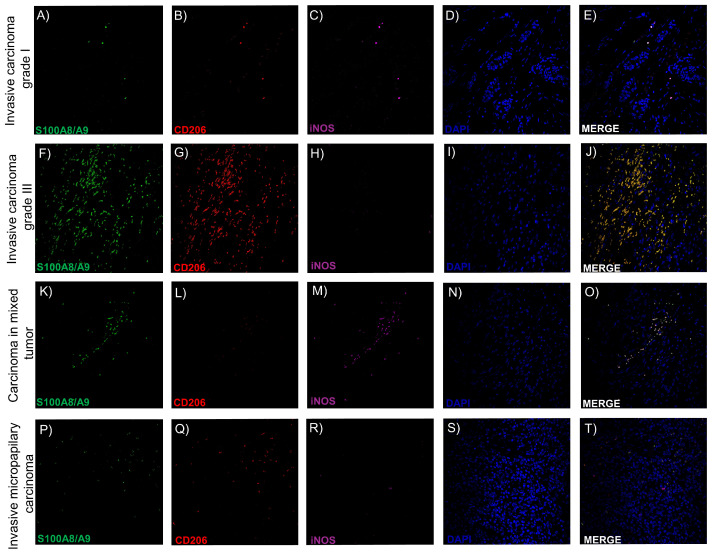
Confocal immunofluorescence staining of intratumoral macrophages in human (**A**–**J**) and canine (**K**–**T**) mammary carcinomas. Nuclei are labeled with DAPI (blue), S100A8/A9 with FITC (green), CD206 with TRITC (red), and iNOS with Cy5 (magenta). Representative images illustrate distinct TAM phenotypes across tumor subtypes with favorable (**A**–**E**,**K**–**O**) and unfavorable (**F**–**J**,**P**–**T**) prognoses. Co-expression patterns reveal four predominant profiles: S100A8/A9^+^/iNOS^–^/CD206^–^, S100A8/A9^+^/iNOS^+^/CD206^–^, S100A8/A9^+^/iNOS^–^/CD206^+^, and S100A8/A9^+^/iNOS^+^/CD206^+^, reflecting phenotypic diversity and immune plasticity within the tumor microenvironment. Images acquired using 40× objective (scale bar = 100 μm) (**A**–**T**).

To explore this hypothesis, we employed multiplex immunofluorescence staining using antibodies against S100A8/A9 (a pan-macrophage and inflammatory marker), iNOS (inducible nitric oxide synthase, indicative of M1 polarization), and CD206 (mannose receptor, a canonical M2 marker), in combination with DAPI nuclear staining. Representative images from human (A–J) and canine (K–T) mammary carcinomas are shown in Figure 5. Tumor subtypes with favorable prognoses (e.g., ICgI and CMT) are shown in panels A–E and K–O, while aggressive subtypes (e.g., ICgIII and SC/IMC) are shown in panels F–J and P–T.

We identified four predominant TAM phenotypes based on marker co-expression: (1) S100A8/A9^+^/iNOS^–^/CD206^–^, (2) S100A8/A9^+^/iNOS^+^/CD206^–^, (3) S100A8/A9^+^/iNOS^–^/ CD206^+^, and (4) S100A8/A9^+^/iNOS^+^/CD206^+^. These patterns suggest that a significant proportion of TAMs do not conform to strictly M1 or M2 classifications but instead adopt mixed or transitional states, reflecting the functional plasticity of macrophages in situ. Particularly in high-grade and invasive tumors, triple-positive TAMs (expressing all three markers) were frequently observed, hinting at a complex immunoregulatory landscape where TAMs may simultaneously support immune evasion, tissue remodeling, and local inflammation.

The detection of these hybrid phenotypes in both species reinforces the biological relevance of comparative oncology models and suggests that simplistic M1/M2 frameworks may be insufficient to describe macrophage function in the tumor microenvironment. Instead, a spectrum-based or context-dependent approach may better capture the nuanced roles of TAMs in cancer progression [76,77]. These findings highlight the need for advanced profiling techniques, such as spatial transcriptomics or single-cell analysis, to further dissect TAM heterogeneity and identify therapeutic targets based on functional rather than binary phenotypes.

## 3. Discussion

In this comparative study involving canine and human mammary carcinomas, we demonstrated that the infiltration of tumor-associated macrophages (TAMs) and the disorganization of collagen fibers are closely correlated with histological subtypes of poor prognosis. In both species, tumors such as invasive micropapillary carcinoma and solid or grade III carcinomas exhibited higher TAM densities, as well as shorter, thicker, and disorganized collagen fibers within the tumor parenchyma. Conversely, low-grade tumors—such as human tubular carcinoma and canine carcinoma in mixed tumors—showed lower macrophage infiltration and longer, more structured collagen fibers with increased anisotropy and reduced angular dispersion.

These findings reinforce the concept that the tumor microenvironment (TME) is a decisive factor in cancer progression. TAM presence and extracellular matrix (ECM) remodeling emerged as key modulators of mammary tumor aggressiveness. The association between disorganized collagen and high macrophage infiltration suggests a potential feedback loop between matrix remodeling and innate immune activation, which may contribute to immune evasion, local invasion, and metastatic spread [25,43,44,48,51,57,78,79,80,81]. Moreover, the parallel histological, immunological, and stromal patterns observed in canine and human samples validate the use of canine models as robust translational platforms.

Previous studies have linked elevated TAM densities with poor prognosis in human breast cancer, particularly in the triple-negative, basal-like, and micropapillary subtypes [42,48,74,82,83]. In veterinary oncology, similar associations have been reported in aggressive canine mammary tumors [50,84]. However, few studies have addressed the coordinated evolution of immune and stromal compartments in both species. Our results expand upon this evidence, providing novel cross-species data that highlight conserved TAM–ECM dynamics.

In addition to supporting the immunosuppressive and pro-angiogenic functions of CD206^+^ macrophages [42,45,83], our findings align with the emerging insights into the mechanotransduction pathways by which ECM stiffness and fiber architecture modulate macrophage recruitment and polarization [52,71,85]. Dense and fragmented collagen matrices are known to trigger macrophage activation via integrin signaling, promoting pro-tumoral phenotypes [71,86]. The presence of shorter and disorganized collagen fibers in our high-TAM samples reinforces this mechanistic link.

The interplay between TAMs and ECM components constitutes a critical axis of tumor progression. In our samples, TAM infiltration was consistently associated with specific ECM configurations, suggesting a bidirectional relationship. TAMs secrete ECM-modifying enzymes, including matrix metalloproteinase-9 (MMP9) and lysyl oxidase (LOX), which contribute to collagen cross-linking and remodeling [84]. These changes increase tissue stiffness and interstitial pressure, both of which facilitate tumor cell dissemination and impair immune surveillance.

Reciprocally, the ECM architecture can influence macrophage behavior. Mechanosensitive pathways such as YAP/TAZ and integrin-mediated cascades modulate macrophage gene expression and phenotypic plasticity [45,72]. Our results support the hypothesis that the ECM and TAMs form a dynamic and reciprocal ecosystem that co-evolves during tumor progression. Therapeutically, this axis could be disrupted using LOX, MMP, or integrin inhibitors, particularly in combination with immunomodulatory agents.

In both canine and human cohorts, high TAM infiltration was significantly associated with reduced overall survival. In dogs, a TAM count ≥ 400 cells was linked to a median survival of only 168 days, compared to 558 days in the low-infiltration group. In the human cohort, patients with ≥250 TAMs had shorter overall survival (log-rank p=0.0171, HR = 0.19). These results underscore the prognostic value of macrophage density across species, extending prior human studies [52,53] and reinforcing the utility of canine models for studying the clinical impact of immune–stromal interactions.

Therapeutic strategies targeting TAMs are rapidly evolving and include agents that block macrophage recruitment (e.g., CCR2 or CSF1R inhibitors), reprogram TAMs toward M1-like anti-tumor phenotypes, or deplete pro-tumoral populations [87,88]. Clinical trials combining TAM-targeted agents with immune checkpoint inhibitors or chemotherapy have yielded promising results. Simultaneously, ECM-targeted interventions such as LOX inhibitors have shown efficacy in preclinical models by reducing metastasis and improving drug delivery [89]. The integration of TAM–ECM profiling into therapeutic stratification may enhance treatment responses in aggressive breast cancer.

The use of naturally occurring canine mammary tumors offers distinct translational advantages. Unlike murine models, which often lack complex stromal interactions and immune competence, dogs develop spontaneous tumors with comparable histological, molecular, and microenvironmental features to those in humans [59,60]. Shared environmental exposures and hormonal contexts further support their relevance. In our study, the similarities between solid carcinoma (dog) and grade III carcinoma (human), as well as between canine mixed tumors and human tubular or grade I carcinomas, permitted meaningful comparisons of TAM distribution and ECM architecture [61,64,65].

Despite the strengths of our comparative approach, limitations should be acknowledged. First, our reliance on FFPE tissues precluded functional assays, such as cytokine profiling or live-cell imaging. Second, the TAM phenotyping was based on a limited panel of markers (S100A8/A9, iNOS, and CD206) that, although validated, may overlap with other myeloid populations. Spatially resolved transcriptomics and multiplexed imaging techniques could improve cell type attribution. Third, the follow-up in the human cohort was incomplete, limiting the resolution of the survival analyses. Nonetheless, the statistically significant association between TAM burden and survival provides a robust signal that warrants further validation in prospective datasets.

Finally, species-specific nuances—such as differences in immune cell subsets, hormonal cycles, and tumor classification schemes—must be considered when translating findings. Integrative multi-omic approaches across species will be essential to refine comparative oncology models and uncover conserved drivers of tumor progression.

**Figure 6:**
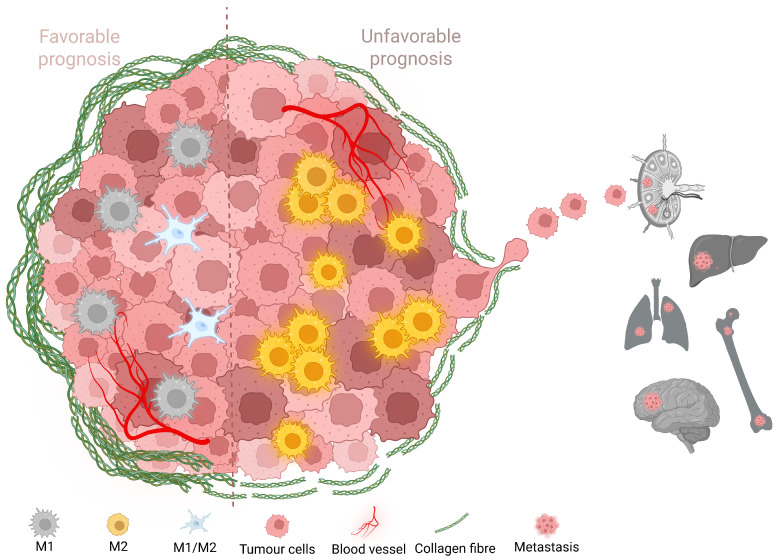
Schematic representation of tumor microenvironments associated with favorable and unfavorable prognostic profiles in mammary carcinomas. Tumors with poor prognoses are characterized by high densities of tumor-associated macrophages (TAMs) exhibiting mixed M1/M2 phenotypes, in addition to shorter disorganized collagen fibers with reduced anisotropy—features linked to extracellular matrix (ECM) remodeling, increased stiffness, and enhanced invasive capacity. Conversely, tumors with favorable prognoses display sparse TAM infiltration and longer well-aligned collagen fibers, which contribute to a more structured ECM and restrained tumor progression. This schematic synthesizes the histological, immunophenotypic, and biomechanical features observed in both canine and human samples, highlighting the interplay between immune and stromal components in defining tumor behavior. Created with BioRender.

Our findings add to the growing recognition of the tumor microenvironment as a critical determinant of cancer progression and therapeutic response. In both human and canine mammary carcinomas, high TAM infiltration was closely associated with collagen fiber disorganization and histological subtypes of poor prognosis. This interplay was visually synthesized in Figure 6, which illustrates the contrasting microenvironmental landscapes of favorable versus aggressive tumors. Disorganized collagen networks and elevated macrophage density appear to foster invasion and metastatic spread, whereas structured ECM architectures and lower TAM burdens are associated with more indolent behavior.

By integrating histopathological, immunophenotypic, and collagen-based imaging analyses in a cross-species model, we provide new insights into conserved tumor–stroma interactions. This multi-dimensional profiling of the TME supports the potential utility of combined TAM and ECM markers for prognostic stratification and microenvironment-targeted interventions in breast cancer.

Looking ahead, future efforts should prioritize multi-parametric spatial mapping, functional dissection of immune–stromal circuits, and the development of therapeutic strategies that reprogram the TME—either by modulating TAM phenotypes or by restoring ECM homeostasis—to synergize with standard treatments.

Taken together, our results reinforce the notion that tumors are not merely cellular proliferations but dynamic tissues shaped by reciprocal interactions between malignant, immune, and stromal components. Deciphering this crosstalk is not only a scientific imperative; it holds promise for transforming clinical oncology.

## 4. Materials and Methods

### 4.1. Ethical Aspects

This retrospective study adhered to the fundamental ethical principles outlined in Brazilian law no. 11.794 (8 October 2008) and decree no. 6.899 (July 2009), as well as the guidelines set forth by the National Council for the Control of Animal Experimentation (CONCEA) and the international ARRIVE guidelines. All experimental protocols involving canine tissues were approved by the Ethics Committee on the Use of Animals at UFMG (protocol number 83/2021). Human tissue research was conducted in accordance with the Declaration of Helsinki and approved by the Research Ethics Committee at UFMG (COEP-UFMG, protocol number 43947521.3.0000.5149/2021). Informed consent was waived by COEP-UFMG due to the retrospective use of archival paraffin-embedded histopathological specimens.

### 4.2. Case Selection

A total of 58 human breast tumor samples and 59 canine mammary tumor samples were retrospectively selected. Canine samples originated from the Comparative Pathology Laboratory archives at UFMG, while human samples were obtained from the biopsy sector of the UFMG Medical School. Only primary mammary carcinoma samples were included in both cohorts; no metastatic lesions were analyzed. Twenty control tissues from healthy canine mammary glands and twenty from human mammary glands were obtained. In dogs, samples were collected from benign areas of the same mammary chain; in women, tissues were sourced from elective breast reduction surgeries.

Selection criteria included complete clinical–pathological annotation (tumor size, histological grade, and presence of regional/distant metastasis), availability of FFPE blocks, and representative tissue architecture. Histological classification of human breast carcinomas followed the most recent World Health Organization (WHO) criteria [61], whereas canine mammary tumors were classified according to the 2019 Consensus on the Diagnosis, Prognosis and Treatment of Canine and Feline Mammary Tumors [64]. For human samples, molecular subtype classification was determined by immunohistochemical profiling of estrogen receptor (ER), progesterone receptor (PR), and HER2 expression, following established guidelines [61]. Canine tumors were immunophenotyped with the same panel to allow comparative molecular stratification [90]. Tumor grading followed the Nottingham Histologic Score (Elston–Ellis modification of the Bloom–Richardson system).

### 4.3. Immunohistochemistry Analysis

Formalin-fixed paraffin-embedded tissue sections (3–5 µm) were dewaxed, rehydrated, and subjected to antigen retrieval using citrate buffer (pH 6.0) under heat-induced conditions (95 °C, 20 min). Endogenous peroxidase was quenched with 3% hydrogen peroxide. Sections were incubated with anti-CD68 antibody (Dako, Santa Clara, CA, USA, 1:100 dilution) overnight at 4 °C. Detection was performed using the EnVision+ Dual Link System-HRP kit (Dako), with DAB as chromogen. Counterstaining was conducted with Harris hematoxylin.

Macrophage quantification was performed by selecting five intratumoral ‘hotspot’ fields under 400× magnification per sample. Fields were pre-selected at 100× to identify the most densely stained areas. Only CD68-positive cells with classical macrophage morphology (large, irregular cytoplasm, and kidney-shaped nucleus) were counted. Observers were blinded to clinical outcomes. Tumors were stratified as high or low infiltration using pre-defined cut-off values within each cohort: 400 cells for canines, based on the median count; and 250 cells for humans, based on the distribution of available cases.

Given the lack of standardized cut-off values for TAM quantification in mammary carcinomas, this data-driven approach allowed internal stratification and hypothesis generation, although it remains exploratory.

### 4.4. Immunofluorescence Analysis

Immunofluorescence (IF) was performed as previously described [63,91]. Tissue sections (5 µm) were deparaffinized, rehydrated, and subjected to antigen retrieval using Trilogy^TM^ solution (Cell Marque, Darmstadt, Germany) at 120 °C for 20 min. Samples were permeabilized in PBS containing 0.2% Triton-X-100 and subsequently blocked with 1% BSA to reduce nonspecific binding.

Primary antibodies included anti-S100A8/A9-FITC (Abcam, Cambridge, UK, clone MAC387, 1:400), anti-iNOS (Santa Cruz Biotechnology Inc., Dallas, TX, USA, polyclonal rabbit, 1:100), and anti-CD206-R-PE (Beckman Coulter, Brea, CA, USA, clone 3.29b1.10, 1:100). For iNOS detection, Alexa Fluor 647-conjugated secondary antibody (goat anti-rabbit, 1:1000, Thermo Fisher, Waltham, MA, USA) was applied following overnight primary incubation at 4 °C. S100A8/A9 and CD206 were incubated for 1 h at room temperature. Nuclei were counterstained with DAPI. Negative controls included omission of primary antibodies. Confocal images were acquired using a Zeiss LSM 880 microscope (Carl Zeiss Microscopy GmbH, Jena, Germany—CAPI platform), with excitation/emission parameters as follows: DAPI (405/415–480 nm), FITC (488/500–525 nm), R-PE (543/550–630 nm), and Alexa 647 (>650 nm).

While S100A8/A9 and iNOS have been widely used to identify tumor-associated macrophage subsets, they are not exclusively expressed by macrophages and may also label other myeloid or inflammatory cells, particularly in the context of tissue damage or immune activation. To reduce misidentification, only cells displaying classical macrophage morphology—irregular cytoplasm and kidney-shaped nucleus—were included, and marker localization was evaluated in relation to nuclear DAPI staining. Nonetheless, we acknowledge that definitive cellular identification requires multiplexed single-cell resolution techniques such as spatial transcriptomics or high-plex imaging.

The selected marker panel captures distinct macrophage activation states. CD206 is a canonical marker of alternatively activated (M2-like) macrophages, often associated with tissue repair, immune regulation, and tumor progression. iNOS marks classically activated (M1-like) macrophages with pro-inflammatory and cytotoxic phenotypes. S100A8/A9, a calcium-binding protein complex, is involved in monocyte/macrophage recruitment and has been implicated in early inflammatory TAM subsets and the establishment of pre-metastatic niches [72,74].

### 4.5. Multiphoton Imaging and SHG Analysis

Second-harmonic-generation (SHG) and two-photon excited fluorescence (TPEF) imaging were conducted on H&E-stained slides using an Olympus FV300/BX61 upright multiphoton microscope (Olympus Corporation, Tokyo, Japan.) Collagen organization was assessed in both normal and neoplastic regions. Per tumor, a minimum of 5 images were acquired from representative intratumoral areas. All image acquisitions were conducted using identical laser power, exposure time, and detector gain settings to ensure consistency across samples. Collagen fibers were analyzed in 512 × 512 pixel fields at 40× magnification, corresponding to 320 µm per field. Images were acquired from tumor centers to minimize edge effects.

Quantitative SHG analysis was performed using PyFibre (Pyfibre Version 2.1.1, open-source, UFMG) Parameters extracted included collagen fiber length, orientation, and density. Segmentation thresholds were defined empirically and uniformly applied to all images. Quality control excluded artefactual fields or those with low signal-to-noise ratios.

### 4.6. Statistical Analysis

Data distribution was tested using the Shapiro–Wilk test. Parametric (ANOVA with Tukey’s HSD) or non-parametric (Kruskal–Wallis with Dunn’s test) methods were used as appropriate. Spearman’s correlation was employed to assess associations between macrophage counts and collagen parameters. Kaplan–Meier survival analyses were conducted for both canine and human cohorts using the log-rank test. TAM infiltration was binarized using cut-offs of 400 (canines) and 250 (humans). Hazard ratios (HRs) and 95% confidence intervals were calculated to assess prognostic impact. Chi-squared or Fisher’s exact tests explored categorical associations. All statistical analyses were performed in GraphPad Prism v8.0; *p*-values were considered significant at *p* < 0.05.

## 5. Conclusions

This comparative study highlights the pivotal role of tumor-associated macrophages (TAMs) and collagen remodeling in shaping the biological behavior and prognosis of mammary carcinomas in both humans and dogs. Our findings demonstrate that high TAM infiltration—particularly of CD206^+^ phenotypes—is associated with increased tumor aggressiveness, shortened survival, and marked alterations in the extracellular matrix (ECM) architecture.

The inverse correlation between macrophage density and collagen fiber length, coupled with enhanced fiber disorganization, underscores the active participation of immune–matrix crosstalk in driving tumor progression. By integrating immunohistochemical, immunofluorescence, and second-harmonic-generation microscopy analyses, we provide a multi-dimensional characterization of the tumor microenvironment across species.

Importantly, the phenotypic plasticity of TAMs, revealed by the concurrent expression of M1 and M2 markers, suggests that tumor-associated inflammation is more complex than the binary paradigm often assumed. This complexity has direct implications for the development of TAM-targeted therapies, which may need to consider macrophage heterogeneity and the microenvironmental context.

Our results reinforce the relevance of spontaneous canine mammary tumors as a powerful translational model for breast cancer. The cross-species parallels observed in the macrophage infiltration patterns, collagen architecture, and prognostic associations support the inclusion of veterinary models in preclinical immuno-oncology research and biomarker discovery.

From a clinical standpoint, the integration of TAM profiling and ECM analysis may enhance tumor stratification beyond conventional histopathological parameters. Future approaches that combine immune modulation with matrix-targeting strategies—such as LOX inhibitors, integrin blockers, or MMP antagonists—hold promise for disrupting tumor–stroma interactions and improving patient outcomes.

Ultimately, our study suggests that dual targeting of TAMs and ECM components may open new avenues for precision oncology. These strategies may be particularly valuable in aggressive tumor subtypes characterized by stromal dysregulation, immune suppression, and poor response to conventional therapies. Further studies incorporating spatial transcriptomics, functional assays, and prospective clinical correlations will be essential to translate these insights into therapeutic innovation.

## Data Availability

The data supporting the findings of this study are available from the corresponding author upon reasonable request. Due to ethical and legal restrictions related to patient confidentiality and the use of archived clinical material, the datasets are not publicly available.
